# Computer Assisted Bone Age Estimation Using Dimensions of Metacarpal Bones and Metacarpophalangeal Joints Based on Neural Network

**DOI:** 10.30476/dentjods.2023.95629.1882

**Published:** 2024-03-01

**Authors:** Abdolaziz Haghnegahdar, Hamid Reza Pakshir, Mojtaba Zandieh, Ilnaz Ghanbari

**Affiliations:** 1 Dept. of Oral and Maxillofacial Radiology, School of Dentistry, Shiraz University of Medical Sciences, Shiraz, Iran; 2 Dept. of Orthodontics, Orthodontic Research Center, School of Dentistry, Shiraz University of Medical Sciences, Shiraz, Iran; 3 Artificial Intelligence, Shiraz University, Shiraz, Iran; 4 Dept. of Oral and Maxillofacial Surgery, School of Dentistry, Shiraz University of Medical Sciences, Shiraz, Iran

**Keywords:** Bone age, Metacarpal bones, Metacarpophalangeal joints, Neural network

## Abstract

**Statement of the Problem:**

Bone age is a more accurate assessment for biologic development than chronological age. The most common method for bone age estimation is using Pyle and Greulich Atlas. Today, computer-based techniques are becoming more favorable among investigators. However, the morphological features in Greulich and Pyle method are difficult to be converted into quantitative measures. During recent years, metacarpal bones and metacarpophalangeal joints dimensions were shown to be highly correlated with skeletal age.

**Purpose:**

In this study, we have evaluated the accuracy and reliability of a trained neural network for bone age estimation with quantitative and recently introduced related data, including chronological age, height, trunk height, weight, metacarpal bones, and metacarpophalangeal joints dimensions.

**Materials and Method:**

In this cross sectional retrospective study, aneural network, using MATLAB, was utilized to determine bone age by employing quantitative features for 304 subjects. To evaluate the accuracy of age estimation software, paired t-test, and inter-class correlation was used.

**Results:**

The difference between the mean bone ages determined by the radiologists and the mean bone ages assessed by the age estimation software was not significant (*p* Value= 0.119 in male subjects and *p*= 0.922 in female subjects). The results from the software and radiologists showed a strong correlation -ICC=0.990 in male subjects and ICC=0.986 in female subjects (*p*< 0.001).

**Conclusion:**

The results have shown an acceptable accuracy in bone age estimation with training neural network and using dimensions of bones and joints.

## Introduction

Bone age estimation, especially compared to chronological age, is a valuable measure in diagnosing, endocrine malfunction, syndromes, and growth disorders among children [ 1 ]. Skeletal age is a more accurate assessment for biologic development [ 2 ]. Skeletal age is an important indicator of final height in long bone deformities, and also to decide growth termination in orthognathic surgeries [ 3 ]. Skeletal age assessment is conducted upon morphological features as maturity indicators in left hand radiographies [ 3 ]. Skeletal age is most commonly evaluated by Greulich and Pyle Atlas of Hand Radiographies (1950) [ 4 ], by which a radiologist compares the patient’s hand radiography with standard radiographies in the atlas, focusing on calcification centers and morphological aspects of hand and wrist bones. The age of the most similar image is considered as the patient’s skeletal age [ 3 ]. This method is quite simple and fast; however, Roch *et al*. [ 5 ] and King *et al*. [ 6 ] have shown this method to be quite subjective, with inter-observer age estimation differences to range from 0.37 to 0.6 years as well as intra-observer age estimation differences to range from 0.25 to 0.96 years. 

In 1975, Tanner and Whitehouse [ 7 ] introduced a more objective approach, by which the summation of the developmental scores of twenty ossification centers determined the bone age. This method is rarely used, as it is complicated and time-consuming.

Today, digital radiographs are substituting conventional radiographies and by this turn of events, computer-based techniques are becoming more favorable among investigators. However, many researchers such as Tanner *et al*. [ 8 - 9 ], Dichause *et al*. [ 10 ], Cao *et al*. [ 11 ] and Pietka *et al*. [ 12 ] have reported the morphological features in Greulich and Pyle method to be difficult to be converted into quantitative measures. This is because there is a great variability in the developmental pattern and there are multiple bones in hand and wrist, which must be considered in bone age assessment.

During recent years, other objective indices have been introduced in left hand radiographies, including 2^nd^ to 5^th^ metacarpal bones and metacarpophalangeal joints dimensions, which are simple linear measurements. These quantitative measures were shown to be highly correlated with skeletal age, and are reliable and accurate indices for bone age estimation [ 13 ].

In 2010, Thodberg *et al*. [ 14 ] introduced a software, which could identify 13 bones in hand radiographies, decide a bone age for each bone, and report the mean of the 13 ages as the patient’s skeletal age. This software is able to outline all the bones in hand radiographies, and is focused on morphological features of the bones not the dimensions of these bony structures [ 14 ]. 

Similarly, in 2005, Zhang *et al*. [ 15 ] introduced computer-assisted diagnosis, which decided the bone age by studying the phalangeal region. This approach also was able to extract morphological features of the desired bones. Computer assisted diagnosis could estimate skeletal age with high accuracy for girls older than 6 and boys older than 8 years of age. Later, in 2007, they designed another software which a new region of interest, which was the carpal bones, to specifically estimate skeletal age in boys younger than 5.5 and girls younger than 4 [ 16 ]. Therefore, not all ages could benefit from this new computer assisted bone age estimation method.

In 2018, Larson *et al*. [ 18 ] presented that an automated model for assessment of bone age based on a convolutional neural network can have an accuracy similar to that of current state-of-the-art automated models by using feature-extraction techniques. Their deep learning neural network based system showed to perform at a level similar to that of a trained human reviewer [ 18 ]. Still their model, similar to the previously designed systems, was based on extracting and analysis of the morphological features of hand radiography skeletal compartments and redirecting them into quantitative measures to estimate bone age [ 18 ].

There has been a growing interest in computerizing bone age estimation; however, all previous designed software was based on the classic method of defining and extracting morphological features in hand radiographs. In our previous study [ 13 ], we introduced new quantitative measures for bone age estimation, including length and width of 2^nd^-5^th^ metacarpal bones as well as the width and thickness of 2^nd^-5^th^ metacarpophalangeal joints. Considering the originally quantitative nature and strong correlation of these indices with skeletal age, in this study we have hypothesized that these values have the ability to substitute the traditional morphological features. Therefore, we have utilized and trained a neural network to estimate bone age with these data, including, chronological age, height, trunk height, weight, the length, and width of 2^nd^-5^th^ metacarpal bones and also width and thickness of 2^nd^-5^th^ metacarpophalangeal joints. 

The purpose of this study was to evaluate the accuracy and reliability of the neural network bone age determination based on originally quantitative data, hand bone and joints dimensions, and comparing these values with other recently introduced computerized systems.

## Materials and Method

For this study, we have used the data we had acquired from our previous study [ 13 ], including left hand radiographs, which were downloaded from Digital Hand Atlas Data Base System [ 17 ]. This system includes 1103 left hand radiographies from children with age ranging from 1 year to 20 years. All children were normally developed and medically, they did not have any diagnosed pathology or trauma in the left hand. The children were categorized in four groups based on their race including Asian, African-American, Hispanic, and Caucasian; they also were subcategorized in male and female groups. These Radiographs are available for education and research. The system also provides each subject’s demographic data, including chronological age, height, trunk height, and weight.

In this study, we enrolled only Asian subjects, 333 digital left hand radiographs were selected and downloaded. The inclusion criteria were decided to be the Asian healthy children (as the data revealed in the website) between the age 3 to 18 years whose left hand radiographies and health data were available online at Digital Hand Atlas Data Base System. The exclusion criteria were low quality radiographies, incomplete demographic data, and children younger than 3 years. Therefore, two cases were excluded because of unacceptable quality of the radiographies and 27 cases were removed since they were chronologically younger than 3 years.

Every radiograph was assessed by two radiologists and the mean estimated skeletal age was determined as the final bone age; the data was documented in details in our previous study [ 13 ].

As the first step, by using Photostudio (version 5.5), the resolution of all radiographs was assessed; they all had the same resolution equal to 250 dpi (dot per inch).

The Adobe Photoshop CS5 Extended (Middle Eastern, version 12) software was utilized for processing the images and measurements. During the processing phase first, the smart sharpening filter, which was set on 500% amount and 5x radius, was applied for better edge detection. Then regarding the resolution of images (250 pixel=25 millimeters) the measurement scale was customized. All the linear data were measured in millimeters.

In order to measure the length and width of the metacarpal bones and width and thickness of metacarpophalangeal joints, the ruler tool was used, which indicated the length of the drawn line as L1. The zoom level was set on 100% for measuring bones length, and on 200% for measuring the width and thickness of joints and bones width.

**Figure 1:**
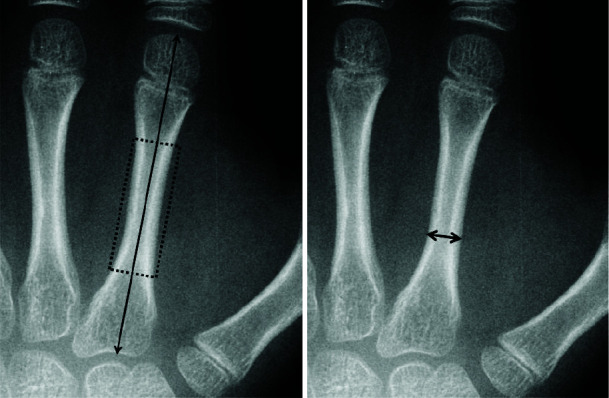
2^nd^ metacarpal bones length and width in hand radiographs

**Figure 2:**
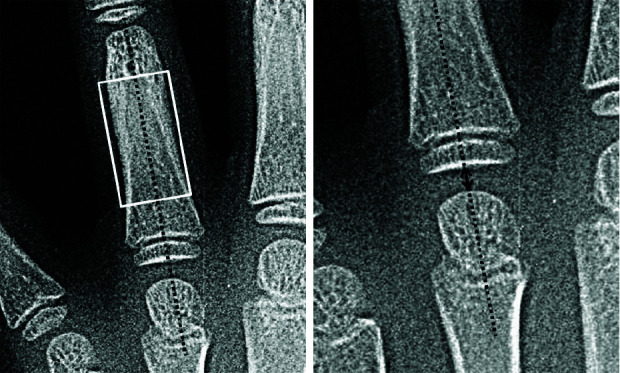
4^th^ metacarpophalangeal joint width in hand radiographs

**Figure 3:**
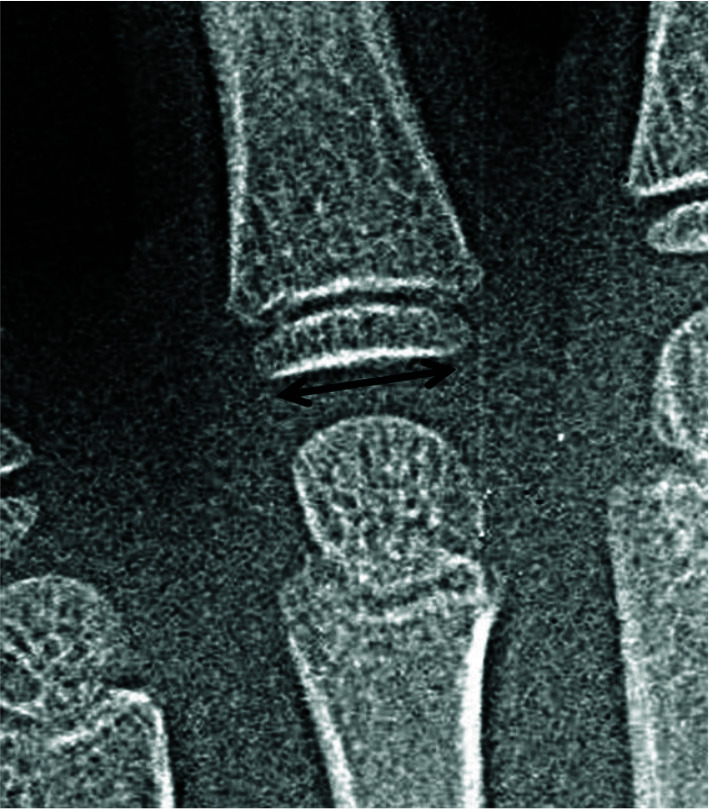
4^th^ metacarpophalangeal joint thickness in hand radiograph

The measuring lines for metacarpal bones length and width are shown in Figure 1. The bone length was measured by the lines parallel with the long axis of the diaphysis region of the bone. The bone width was measured in its narrowest area of diaphysis. Figures 2 and 3 show the measuring lines for metacarpophalangeal joints width and thickness. The joint space width was measured with a line parallel with the long axis of the diaphysis of adjacent proximal phalangeal bone.

Eventually, 21 quantitative features were recorded for each subject including bone age, chronological age, height, trunk height, weight, length, and width of 2^nd^-5^th^ metacarpal bones and width and thickness of 2^nd^-5^th^ metacarpophalangeal joints. The data for each subject were manually entered and saved in two Microsoft Office Excel Worksheets separately for male and female subjects. Each row demonstrated one subject’s data and each column were related to one feature only.

A multilayer perception neural network, using MATLAB, Version 9.10.0 (R2021a) (Natick, Massachusetts, United States: The Math Works Inc., consisting 3 layers, 10 neurons in input layer, 10 neurons in hidden layer and 16 neurons in output layer was utilized to determine bone age, using other 20 quantitative features for each subject. Microsoft Office Excel Work sheets were used for both importing and exporting data.

Since boys show a different growth pattern from girls, male and female subjects were studied separately by two different neural networks, which were designed similarly but were trained and tested separately.

The neural network chose 80% of the subjects randomly for training and leaves the remaining 20% for test process. First for training the age estimation software, an Excel file, which contained all 21 features for each subject, was used. The neural network automatically tries to find a correlation between the first 20 features and the last feature, which is the bone age.

Then, for testing the neural network, another Excel file was prepared which was exactly the same as the first one, except that this file contained 20 features and did not include the last column which was the bone ages of the subjects. The trained neural network determined each subject’s bone age and exhibited the assessed skeletal ages sequentially in another excel file.

To evaluate the accuracy and reliability of the age estimation software, SPSS (version 26), paired t-test, and inter-class correlation was used, comparing the age estimated by the radiologists with the age determined by the neural network.

## Results

The hand radiographs of 155 female subjects (50.99%) and 149 male subjects (49.01%) were evaluated in this study. The chronological age of female subjects ranged 3 to 19 years (mean=11.96); this rage was 4 to 19 years (mean=12.27) for male subjects.

Tables 1 to 4 show the paired p-test results, which compares the difference between the mean bone ages determined by the radiologists and the mean bone ages assessed by the neural network. The differences were not statistically significant (*p*= 0.119 in male subjects and *p*= 0.922 in female subjects).

**Table 1:** Paired Samples Statistics in male subjects

	Mean	N	Std. Deviation	Std. Error Mean
PairBone Age assessed by radiologists	12.75752	149	4.317994	0.353744
Age Estimation Software Results	12.74925	149	4.356785	0.356922

**Table 2:** Paired Samples Results in male subjects

	Paired Differences					*p* Value
	Mean	Std. Deviation	Std. Error Mean	95% Confidence Interval of the Difference		
				Lower	Upper	
PairBone Age	0.008268	0.850727	0.069694	-0.129456	0.145993	0.119
A.E. Software Results						

**Table 3:** Paired Samples Statistics in female subjects

	Mean	N	Std. Deviation	Std. Error Mean
PairBone Age assessed by radiologists	12.2112	155	4.43393	0.35614
Age Estimation Software Results	12.1352	155	4.29582	0.34505

**Table 4:** Paired Samples Results in female subjects

	Paired Differences					*p* Value
	Mean	Std. Deviation	Std. Error Mean	95% Confidence Interval of the Difference		
				Lower	Upper	
PairBone Age	0.075994	1.026158	0.082423	-0.086832	0.238819	0.922
A.E. Software Results						

Tables 5 and 6 show the inter-class correlation between the bone ages assessed by the radiologists and the bone ages determined by the neural network. There was a significant relationship between the bone age determinations (*p*< 0.001). The results obtained from software and the radiologists showed a strong correlation (ICC= 0.990 in male subjects and ICC= 0.986 in female subjects). The descriptive statistics are also demonstrated in tables 7 and 8.

**Table 5:** Inter-Class Correlation in male subjects

	Interclass Correlation	95% Confidence Interval	
		Lower Bound	Upper Bound
Single Measures	0.981	0.974	0.986
Average Measures	0.990	0.987	0.993

**Table 6:** Inter-Class Correlation in female subjects

	Interclass Correlation	95% Confidence Interval	
		Lower Bound	Upper Bound
Single Measures	0.972	0.962	0.980
Average Measures	0.986	0.981	0.990

**Table 7:** Absolute Value of the Difference in male subjects

	N	Minimum	Maximum	Mean	Std. Deviation
Absolute Value of the Difference	149	0.001	3.261	0.65298	0.542715
Valid N	149				

**Table 8:** Absolute Value of the Difference in female subjects

	N	Minimum	Maximum	Mean	Std. Deviation
Absolute Value of the Difference	155	0.008	3.025	0.82553	0.610642
Valid N	155				

## Discussion

In our previous study, we have introduced 16 new quantitative indices Forskel *et al*. age estimation. These measurements were proven to be highly correlated with skeletal age and could be utilized to determine bone age [ 13 ].

Currently, there have been many researches and commercially-introduced software regarding image processing and data extraction, which can measure dimensions of bones and joints [ 1 , 12 , 15 , 19 ]. In this study, we have only tried to computerize the last steps in skeletal age estimation, which is analyzing these acquired data to estimate bone age. 

Regarding the results of this study, neural network is an accurate and reliable program for bone age estimation, analyzing the dimensions of metacarpal bones and metacarpophalangeal joint spaces. Concerning the accuracy of neural network age estimations, there was a very strong correlation between the estimated bone age by the neural network and the radiologists (0.990 in male subjects and 0.986 in female subjects). In estimations for male and female subjects, the analysis could not show any statistical difference between neural network results compared with the radiologists’ observations (*p*= 0.119 in male subjects and *p*= 0.922 in female subjects).

Moreover, regarding the reliability of the age estimated by neural network, the mean of absolute value of the difference of the estimated age by the neural network compared with the radiologists is 0.65 years in male subjects and 0.83 years in female subjects. These differences are comparable with Pyle and Greulich method, with inter-observer age estimation differences, which have been reported to range from 0.37 to 0.6 years, and intra-observer age estimation differences to range from 0.25 to 0.96 years in the classic method [ 5 - 6 ]. Therefore, training a neural network with skeletal dimensions can be considered as an accurate and reliable computer-based automatic bone age estimation method, specifically when compared with the traditional method of using an atlas by a radiologist.

Old methods of bone age determinations include morphological features, and it is quite difficult for software to be trained to extract such data. Since the method of analyzing morphological indices has even shown a noticeable inter- and intra- observer differences even when used by radiologists, morphological features may not be the best choice for computer assisted skeletal age estimation. As an alternative, the dimensions of bone and joints can easily be measured with digital image processing software, either specifically designed for hand radiographs or other more common software like Photoshop and so on with the least inter- intra observer differences.

Pyle and Greulich method is still the most common method both used and taught for bone age estimation around the world, yet the shortcomings of this method can be encountered as being time consuming, based on morphological features of carpal bones and traditionally utilizing an atlas by a radiologist with the least employment of computerized software.

With the advent of digital radiology, an interest started to develop in the area of computerizing the diagnosis and evaluations of radiographies. The pioneers of this concept in skeletal age estimation were Michael *et al*. [ 19 ] and Pietka *et al*. [ 20 ]. In 1989, Michael *et al*. [ 19 ] designed and introduced a program in which, specific bones were identified manually by a user, outlined by the software, and quantitative values were extracted based on the morphological features of each bone. These values were interpreted into skeletal age. In 1991, Pietka *et al*. [ 20 ] designed a bone age estimation program, based on manual detection of phalangeal bones, and computer-based analysis of the morphological features of these regions, and eventually extracting features, which could further be rendered as skeletal age. Both programs were more focused on recognition, segmentation, and outline detection of the specific bony structures in digital images.

In 1994, Tanner *et al*. [ 8 ] introduced a completely new semi-computerized method for skeletal age estimation (Tanner-Whitehouse) by which a developmental value was given to each bony structure in a hand radiograph. For detecting each bone, the hand radiograph was superimposed on a computerized template during scanning the conventional hand radiography films. This new method showed a higher reliability and lower intra-observer differences in bone age assessment in compare with manual method of Greulich and Pyle [ 9 ].

However, the main disadvantage of all these primary efforts for computerizing the analysis was being more time consuming than manual readings by radiologists.

Zhang *et al*. [ 15 ] in 2005, introduced computer assisted diagnosis, which detected the bone age by studying the morphological features of phalangeal region. This software could estimate skeletal age with high accuracy for girls older than 6 and boys older than 8 years of age. In 2007, they designed another software to specifically estimate skeletal age in boys younger than 5.5 and girls younger than 4 by focusing on morphological features of the carpal bones. Their method failed to include all range of age, however with the two areas of interest in each range, the software had a high accuracy in age estimation [ 16 ]. 

In our study, with measuring dimensions of skeletal and articular compartments and computerizing the analysis with neural network, all ages between 3 to 18 years could be included for bone age estimation with one method, with high accuracy and reliability.

The first fully automated commercially available system for skeletal age assessment (BoneXpert; Visiana Aps, Holte, Denmark, available at http://www.bonexpert.com) was first designed and introduced by Van Rijn and Thodberg [ 21 ] in 2013. This software utilizes a feature-extraction technique that extracts and reconstructs the borders of the bones in hand radiographs. This system is now available and clear to use clinically in Europe. The designers evaluated the accuracy of the model and reported that the mean standard deviation in the differences between the BoneXpert model and manual assessments ranged from 0.55 to 0.76 years, with a weighted average of approximately 0.68 years. In our study, the mean of absolute value of the difference of the estimated age by the neural network compared with the radiologists was 0.65 years in male subjects and 0.83 years in female subjects. Therefore, in compare with BoneXpert, the neural network based analysis of skeletal dimensions shows a promising validity, accuracy, and reliability as a computerized system for bone age assessment. 

Most recently, in 2018, Larson *et al*. [ 18 ] also presented deep learning neural network system for bone age estimation based on morphological features of hand skeleton. They compared the bone age estimated by the neural network deep learning system with the bone ages assessed by three radiologists. Their study results showed that the mean bone age estimated by the model was not significantly different from that estimated by any of the reviewers (p Values = 0.34, 0.36, 0.57).Their study concluded that their deep learning–based model performs at a level similar to that of a trained human reviewer. Similarly in this study, we have shown no significant difference between the age estimated by the software and the bone age determined by the radiologists (*p*= 0.119 in male subjects and *p*= 0.922 in female subjects).

Moreover, when Larson *et al*. [ 18 ] compared the performance of the model with the mean of the reviewer estimates, the mean absolute difference in bone age estimates was 0.50 years. Similarly, in this study, we have shown the mean absolute difference between the system and radiologists to be 0.65 and 0.82 in male and female subjects, respectively. Similar to Larson *et al*.’s neural network [ 18 ], our neural network has demonstrated an acceptable accuracy in skeletal age determination in compare with the classic method of Greulich and Pyle, which has been reported with intra-observer age estimation differences even up to 0.96 years [ 5 - 6 ].

Since skeletal age estimation only includes physiological changes and excludes abnormalities, especially in morphology and dimensions, we believe that analysis of the data is completely achievable by a neural network system.

Apart from the promising results of this study, one of the significant limitations of this study was being focused on Asian children. In the future studies, we recommend evaluation of correlation between bone and joint compartments dimensions with skeletal age in other ethnicities including Caucasian, African, and Hispanic children, or specific regions or countries. Subsequently, if the results of correlation were strong enough, designing, and training deep learning neural networks for increasing the availability of these newly introduced measurements in assessing skeletal age would be a first-rate proposal. As we have previously demonstrated a strong correlation between skeletal age and bone and joint dimensions in hand radiographies, it is most rational to extract and use these data through a computer-based system, to decrease errors, be able to utilize all these valuable measurements and reduce the time consumed for radiography readings. 

The results have shown an acceptable accuracy in bone age estimation with training neural network and using dimensions of bones and joints. Nowadays, a neural network is the most commonly used data analyzing application for solving artificial intelligence problems. Using this vessel for bone age estimation has shown promising results and further application of the bone and joint dimensions together with development of a neural network can be a new computer-based method for skeletal age determination.

## Conclusion

This new automatic neural network based system has shown reliable and accurate skeletal age estimations based on dimensions of skeletal structures rather than morphological features, and seems to enhance efficiency without compromising the diagnostic accuracy.
